# Unusual Synchronous Arbitrary‐Gate Doppler Spectra Enable Intraoperative Hemodynamic Warning of Cerebral Hyperperfusion Syndrome on Moyamoya Disease

**DOI:** 10.1002/cns.70829

**Published:** 2026-03-11

**Authors:** Xiandi Zhang, Wei Ni, Xing Hu, Heng Yang, Jiabin Su, Hanqiang Jiang, Chao Gao, Ruiyuan Weng, Zhen Fan, Yiming Li, Jinhua Yu, Zhaoling Lu, Yuxiang Gu, Hong Ding

**Affiliations:** ^1^ Department of Ultrasound Huashan Hospital, Fudan University Shanghai China; ^2^ Department of Neurosurgery Huashan Hospital, Fudan University Shanghai China; ^3^ School of Information Science and Technology Fudan University Shanghai China; ^4^ Mindray, MIS Innovation Center Milpitas California USA; ^5^ National Clinical Research Center for Aging and Medicine Fudan University Shanghai China

**Keywords:** cerebral hyperperfusion syndrome, hemodynamics, moyamoya disease, revascularization, synchronous arbitrary gate spectral power Doppler

## Abstract

**Background:**

Cerebral hyperperfusion syndrome (CHS) is a serious complication following revascularization in moyamoya disease (MMD) patients, yet reliable predictors remain scarce. This study aims to evaluate subcortical hemodynamics intraoperatively using a novel ultrasound imaging technique, synchronous arbitrary gate spectral Doppler (SAGSD), and to identify indicators associated with CHS.

**Methods:**

A total of thirty adult MMD patients undergoing revascularization were included. Intraoperative SAGSD imaging, conventional Doppler ultrasound, and indocyanine green videoangiography (ICG‐VA) were performed to assess subcortical hemodynamics at multiple sites before and after anastomosis.

**Results:**

All thirty patients underwent revascularization, and five of the six CHS patients exhibited a distinctive “mountain” sign on SAGSD, characterized by three or more discrete velocity peaks within a single cardiac cycle observed simultaneously across two or more sampling sites. The CHS patients showed a significant increase in velocity‐time integral (VTI) change (median Δ + 0.48 cm [95% CI, 0.31 to 0.65], *p* < 0.001) post‐anastomosis on SAGSD, while conventional Doppler showed no significant change in flow velocity (all *p* > 0.05). Notably, in CHS patients, ICG‐VA showed no statistical difference in flow velocity, delay, and time to peak after anastomosis (all *p* > 0.05), whereas SAGSD demonstrated hemodynamic changes such as VTI (median Δ + 0.67 cm [95% CI, 0.43–0.90], *p* < 0.001) via Doppler spectrum.

**Conclusion:**

SAGSD enables highly sensitive, multi‐position, and contrast‐free hemodynamic evaluation of deep cerebral microvasculature. The “mountain” sign, a novel spectral morphology corroborated by quantitative VTI elevation, is a specific intraoperative biomarker strongly associated with CHS, offering a potential predictor for early intervention and improved surgical outcomes.

## Graphical abstract

Synchronous arbitrary‐gate spectral Doppler (SAGSD) enables real‐time multi‐site hemodynamic monitoring during moyamoya disease revascularization. The novel “mountain” sign, defined as three or more velocity peaks within one cardiac cycle across multiple subcortical sites, was detected in 5 of 6 patients who developed cerebral hyperperfusion syndrome, serving as an early warning indicator for this serious complication, potentially improving intraoperative surveillance in MMD revascularization.

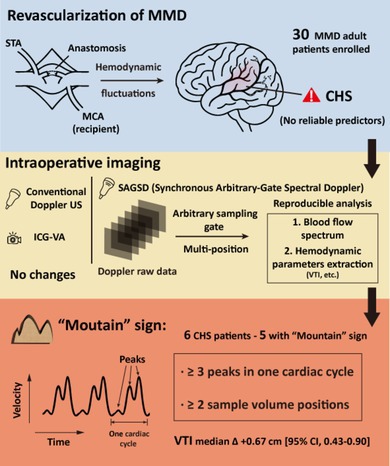

**Abbreviations**
CHS: cerebral hyperperfusion syndrome
CTP: computed tomography perfusion
DSA: digital subtraction angiography
EDV: end‐diastolic velocity
ICG‐VA: indocyanine green videoangiography
MMD: moyamoya disease
PI: pulsatility index
PSV: peak systolic velocity
RBCBS: red‐blood‐cell backscatter
RI: resistive index
S/D: systolic‐to‐diastolic ratio
SAGSD: synchronous arbitrary‐gate spectral Doppler
STA‐MCA: superficial temporal artery to middle cerebral artery
TAMean: time‐averaged mean velocity
UMA: ultra‐microangiography
US: ultrasound
VTI: velocity‐time integral
WSS: wall shear stress

## 1 Introduction

Revascularization, specifically direct superficial temporal artery to middle cerebral artery (STA‐MCA) bypass grafting, has been shown to be beneficial for the prevention of ischemic stroke or transient ischemic attack in patients with moyamoya disease (MMD) [1, 2, 3, 4]. Despite the high precision and technical excellence of bypass surgery, a significant limitation of this procedure is the relatively high incidence of postoperative cerebral hyperperfusion syndrome (CHS) [5]. CHS is characterized by a constellation of clinical symptoms, including headache, seizures, altered mental status, and hemorrhage, accompanied by radiological evidence such as focal hyperperfusion on computed tomography perfusion (CTP) imaging [6, 7, 8]. While these imaging results can only confirm the diagnosis of CHS after the onset of the disease and do not provide effective intraoperative approaches to indicate CHS before its onset. Still, the current clinical guidelines do not specify corresponding technical approaches for the detection of CHS [3, 8, 9].

The prevailing mechanism of CHS posits an impairment of cerebrovascular autoregulation [10, 11, 12]. Sudden post‐bypass hemodynamic shifts drive the dilation of small resistance vessels, precipitating vasogenic edema and, in severe cases, intracranial hemorrhage [13, 14, 15, 16, 17]. Intraoperative indocyanine green videoangiography (ICG‐VA) provides real‐time images of regional perfusion and assesses bypass graft patency by offering functional information, including blood flow direction, velocity, and perfusion area [10, 11]. However, ICG‐VA is limited to cortical‐surface perfusion and cannot clearly show deep anatomy, especially when obscured by normal brain tissue. The subcortical parenchymal hemodynamics, which provide richer cerebrovascular information, remain largely unexplored [6, 12, 18, 19, 20, 21, 22]. Thus, there is an urgent need for an intraoperative real‐time detection method to assess the hemodynamic changes in deep brain regions after revascularization.

**FIGURE 1:**
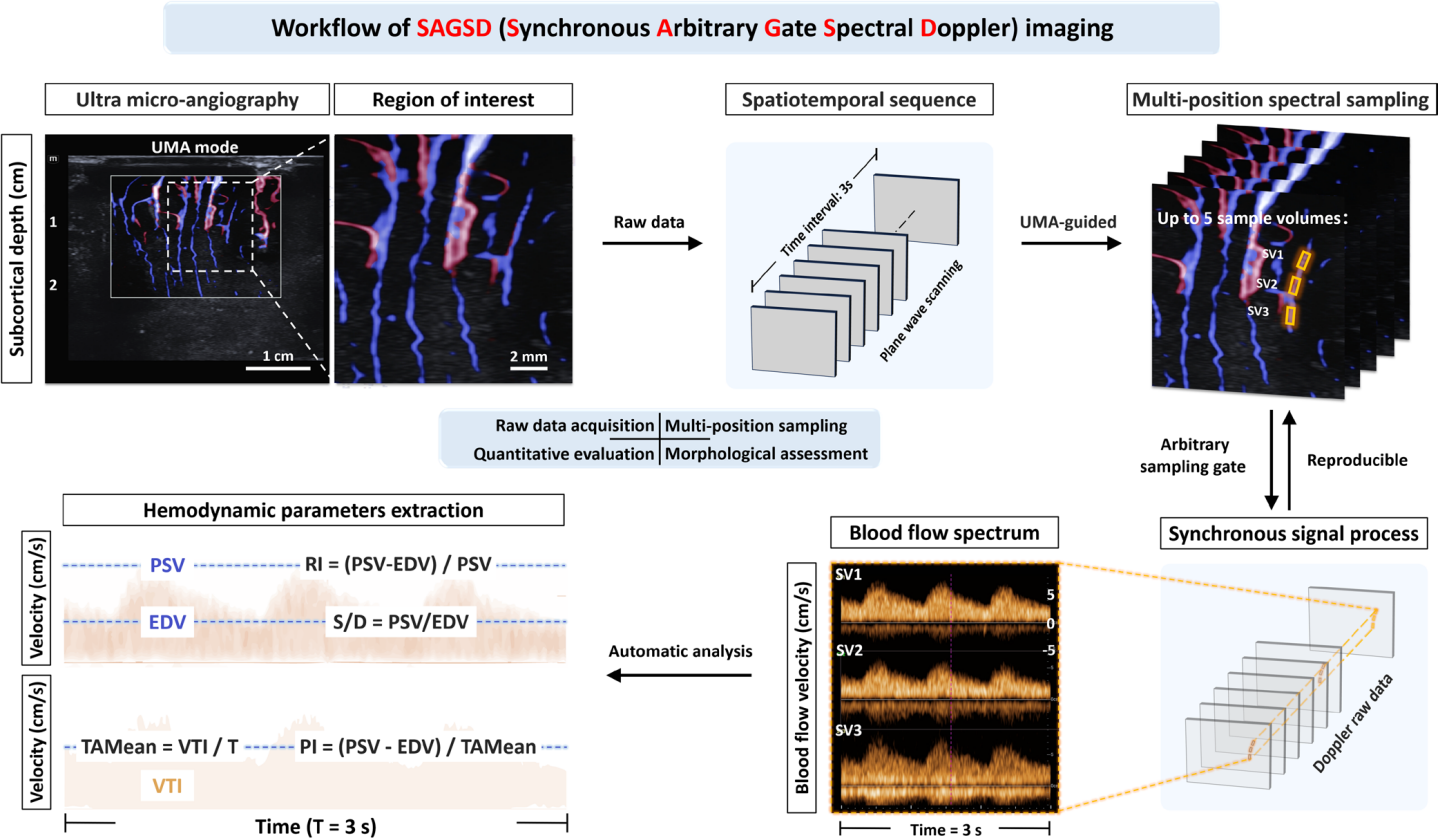
Overview of SAGSD technology and hemodynamic parameter calculation. Schematic of SAGSD imaging workflow, including raw data acquisition, multi‐position sampling, morphological assessment, and quantitative evaluation of hemodynamics. SAGSD, synchronous arbitrary‐gate spectral Doppler; PSV, peak systolic velocity; EDV, end‐diastolic velocity; VTI, velocity‐time integral; TAMean, time‐averaged mean velocity; PI, pulsatility index; RI, resistive index; S/D, systolic‐to‐diastolic ratio.

Conventional Doppler ultrasound (US) enables noninvasive imaging of deep brain tissue during surgery [23], yet its effectiveness in hemodynamic analysis is constrained by the single sampling frame per vessel and limited sensitivity for small‐vessel detection (defined as those with a diameter less than 1 mm) [24]. Additionally, the rich blood supply and numerous small vessels in the cerebral parenchyma complicate consistent identification and multiple measurements of the same small vessel across sonographic sections [25]. Ultra‐microangiography (UMA) exhibits high sensitivity in micro‐vascular detection via high frame‐rate plane wave imaging and wall filtering algorithm [26]. On the basis of UMA technology, we herein introduce a novel modality, synchronous arbitrary‐gate spectral Doppler (SAGSD) imaging, which simultaneously acquires repeatable, temporally registered spectrograms within a unified spatiotemporal framework, enabling sensitive hemodynamic assessment of subcortical small vessels (Figure 1). When combined with UMA, this modality allowed for reproducible, simultaneous, multi‐positional spectral analysis of each detected small vessel within the same temporal window. UMA, based on plane wave signals, primarily serves to assist in localizing small vessels, which is crucial for accurate spectral analysis. Leveraging continuous spatiotemporal Doppler raw data, this approach enabled up to five sampling volumes to be analyzed within an automatically saved 3‐s temporal window, yielding comprehensive hemodynamic profiling intraoperatively.

We hypothesize that SAGSD provides high sensitivity for detecting hemodynamic alterations in MMD patients after revascularization, particularly those who develop CHS. We further sought to identify an intraoperative biomarker that is both visually apparent and quantitatively grounded in hemodynamic data to provide early warning of impending CHS.

## 2 Methods

### 2.1 Selection and Outcome of Patients

This study protocol was approved by our ethics institutional review board and registered at ClinicalTrials.gov, in accordance with the ethical principles of Good Clinical Practice and the Declaration of Helsinki. The consecutive patients with MMD confirmed by digital subtraction angiography (DSA) were admitted as candidates for cerebral revascularization in the neurosurgical ward from March 2025 to July 2025. Written informed consent was obtained from each patient prior to the procedure, and patient privacy was meticulously maintained throughout the study. The inclusion criteria for this study were as follows: The patients who (a) were aged 18 or more; (b) were eligible for revascularization surgery; (c) had no other medical history such as significant traumatic brain injury, brain tumor, epilepsy, neuroinflammatory disease, cerebral metabolic disease, or other severe medical problems. All enrolled patients underwent STA‐MCA anastomosis for revascularization. The exclusion criteria were as follows: (a) Patients who had US imaging with poor quality that was unable to conduct hemodynamic analysis; (b) an incomplete series of intraoperative or postoperative imaging.

Postoperative monitoring included the observation of any new neurological symptoms within seven days after surgery in patients. If such symptoms emerge, imaging examinations should be conducted to determine whether CHS has occurred. CHS was diagnosed based on the presence of typical clinical symptoms (headache, seizures, focal neurological deficits, or altered consciousness) occurring within 14 days post‐revascularization, combined with radiological evidence of focal hyperperfusion on postoperative CTP imaging (cerebral blood flow [CBF] increase > 100% compared with the contralateral side or preoperative baseline in the ipsilateral territory) [27]. Within two weeks after surgery, if the patient develops new symptoms or the original symptoms worsen, a cranial computed tomography or magnetic resonance imaging would be performed to exclude cerebral hemorrhage or cerebral infarction. Based on clinical symptoms and imaging findings, enrolled patients were then divided into CHS and non‐CHS groups.

### 2.2 Surgical Procedures and Perioperative Management

The direct revascularization (anastomosis between the posterior branch of STA and the cortical branch of MCA) was performed by a surgeon under general anesthesia with intravenous propofol and remifentanil. Of note, the distal part of the donor (mostly STA posterior branch) is cut in a fishmouth manner to increase its opening diameter. The opening on the cortical recipient (M4 branch) is performed via a linear arteriotomy: The length of the linear arteriotomy should be at least 2.5 times the size of the diameter of the recipient [28]. During the postoperative period, patients were normohydrated, and their hemoglobin levels were kept between 10 g/dL and 11 g/dL. Blood pressure was maintained at the preoperative level±10 mmHg with a systemic blood pressure less than 140 mmHg. After the operation, patients remain in the intensive care unit for the first night; thereafter, they are moved to the intermediate care unit and finally the ward.

### 2.3 Intraoperative Imaging Process and Image Analysis

Intraoperative ICG‐VA (Carl Zeiss AG, Oberkochen, Germany), SAGSD, and conventional Doppler US were acquired before and after bypass anastomosis; each patient was studied with the L10‐3 probe (Nuewa A20W, Mindray Bio‐Medical Electronics Co. Ltd., Shenzhen, China). The intraoperative imaging examination process is illustrated in Figure S1. After anastomosis, all intraoperative imaging examinations were conducted immediately and completed within 10 min. The detailed setting parameters of SAGSD imaging and conventional Doppler US are provided in the Table S1. For each patient, three SAGSD cine loops were acquired in the region adjacent to the recipient vessel on the surface of the temporal lobe. The ultrasound scanning area was maintained as consistently as possible before and after anastomosis. Each cine loop, lasting 3 s, was independently assessed by two board‐certified radiologists on the day of surgery (immediately postoperatively, prior to the emergence of any clinical symptoms), both of whom had more than 5 years of experience in Doppler ultrasonography, to quantify the hemodynamics of intraparenchymal small vessels (defined as those with a diameter of 0.5 to 1.0 mm and located less than 3 cm beneath the cortical surface). Only vessels oriented nearly perpendicular to the probe axis were interrogated for spectral Doppler acquisition. Both reviewers were blinded to clinical data, perioperative imaging reports, and each other's findings. Inter‐observer reliability of morphological assessments was evaluated using Cohen's kappa test.

### 2.4 Acquisition of Hemodynamic Parameters

To ensure reproducible and representative sampling of subcortical microvasculature, we adopted a standardized protocol for SAGSD hemodynamic parameters, which includes. In the super UMA imaging mode, sampling volumes were strategically placed on small vessels located 0.5–1.5 cm below the brain surface, thereby avoiding major vessels with a diameter of more than 2.0 mm. These small vessels, defined as those with a diameter of 0.5–1.0 mm, are typically small tertiary branches originating from the MCA. For each participant, three consecutive sampling volumes (gate size is between 0.1 and 0.3 mm each) were sampled. The depths of sampling volumes remained approximately the same before and after anastomosis. All parameters were acquired three times under the same technical conditions; the arithmetic mean of the three measurements was entered into the final dataset. The measurement of blood flow velocity using conventional Doppler US was also based on the above process. The calculation method of hemodynamic parameters (peak systolic velocity [PSV], end‐diastolic velocity [EDV], velocity‐time integral [VTI], time‐averaged mean velocity [TAMean], pulsatility index [PI], and systolic‐to‐diastolic ratio [S/D]) based on the SAGSD spectrum was illustrated in Figure 1. The detailed definitions of all hemodynamic parameters were provided in Table S2. To further describe the changes in hemodynamic parameters, the symbol “Δ (delta)” was used to express the difference between the same hemodynamic parameters before and after anastomosis.

The intensity of red‐blood‐cell backscatter (RBCBS) was then extracted from Doppler raw data of SAGSD datasets. Briefly, leveraging the Doppler raw data acquired with SAGSD, RBCBS intensities were computed at each time point coinciding with the distinct peaks on the velocity‐time spectra of the corresponding sample volumes.

The hemodynamic parameters of ICG‐VA, including blood flow velocity, time to peak, and delay, were calculated by FLOW‐800 software (Carl Zeiss AG, Oberkochen, Germany) based on the time‐intensity curve. A total of eight ROIs were randomly selected in each analysis, and the average was taken after removing the highest and lowest values to reflect the overall hemodynamics.

### 2.5 Statistical Analysis

Power analysis was performed using statsmodels in Python (Version 3.14.2) and cross‐validated with G*Power 3.1, and other statistical analyses were performed with SPSS 29.0 (IBM, Armonk, NY) and GraphPad Prism 10.2.0 (GraphPad Software Inc., USA). All clinical data are presented as numbers (%) for categorical variables, and continuous variables are presented as median (interquartile range [IQR]) or mean ± SD. Comparisons between groups were performed using the chi‐square test or Fisher's exact test for categorical variables, and paired *t*‐test or unpaired *t*‐test with Welch correction for continuous variables. Two‐tailed *p* < 0.05 was considered statistically significant.

## 3 Results

### 3.1 Baseline Characteristics of Selective Patients

**FIGURE 2:**
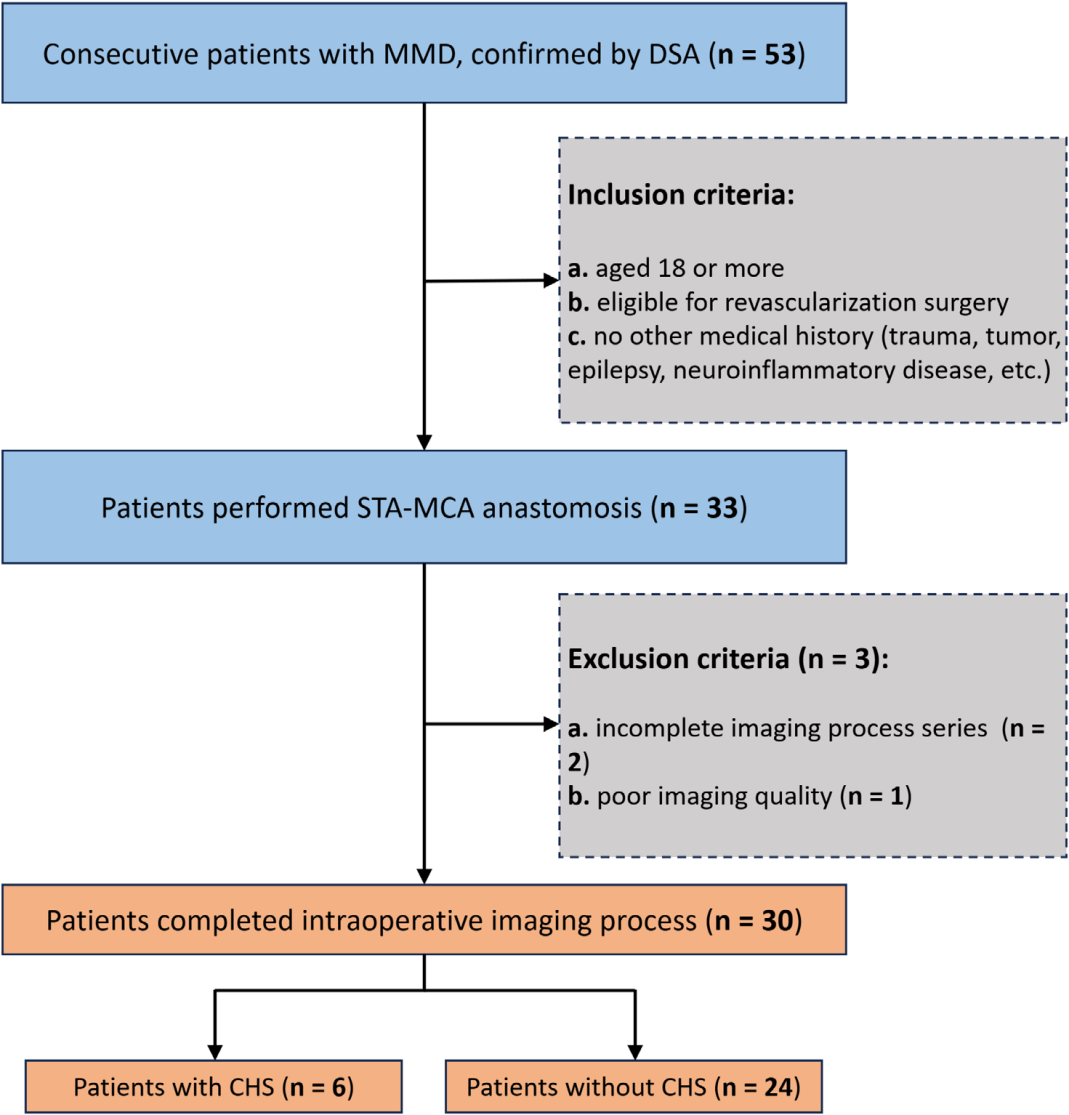
Flow chart of study population enrollment. MMD, moyamoya disease; DSA, digital subtraction angiography; STA‐MCA, superficial temporal artery to middle cerebral artery; CHS, cerebral hyperperfusion syndrome.

Thirty‐three of fifty‐three consecutive patients with a definitive MMD diagnosis by DSA underwent craniotomy for revascularization. After applying exclusion criteria (three patients excluded: Two for incomplete imaging series and one for poor image quality), the remaining thirty adult MMD patients were finally enrolled in the study (Figure 2). In postoperative clinical observation, six patients exhibited symptoms such as severe headache, neurological deficits, and epileptic seizures. Imaging results confirmed the diagnosis of CHS in these patients.

All thirty patients had a median age of 49.4 (IQR, 44.5–55.3) years, and consisted of males (40.0%) and females (60.0%). No significant differences were observed between patients with post‐operative CHS (*n* = 6, 20.0%) and those without CHS (*n* = 24, 80.0%) in terms of age, sex, Suzuki stage, history of stroke, smoking status, body mass index (BMI), diabetes, alcohol consumption, pre‐operative antiplatelet therapy, modified Rankin Score at admission and discharge, hyperlipidemia, hypertension, or involvement of posterior cortical atrophy (PCA) (all *p* > 0.050). Table 1 summarizes the demographic and clinical characteristics of the enrolled MMD patients. Furthermore, the baseline of SAGSD hemodynamic parameters showed no significant difference (all *p* > 0.050) between patients with or without CHS (Table 2).

**TABLE 1:** Clinical Characteristics of Enrolled Patients.

Characteristics	All MMD Patients (*n* = 30)	Patients without CHS (*n* = 24)	Patients with CHS (*n* = 6)	*p‐* value^a^
Age (years) median (IQR)	49.4 (44.5, 55.3)	50.2 (45.0, 55.8)	46.5 (36.5, 54.0)	0.447
Males, *n* (%)	12 (40.0)	11 (45.8)	1 (16.7)	0.358
Previous stroke, *n* (%)	10 (33.3)	8 (33.3)	2 (33.3)	0.694
Smoking, *n* (%)	9 (30.0)	8 (33.3)	1 (16.7)	0.400
BMI^b^, median (IQR)	23.1 (20.9, 25.2)	23.3 (21.0, 25.3)	22.6 (19.9, 25.1)	0.609
mRS at admission, mean ± SD	1.27 ± 0.45	1.25 ± 0.44	1.17 ± 0.408	0.671
Diabetes, *n* (%)	5 (16.7)	3 (12.5)	2 (33.3)	0.254
Alcohol, *n* (%)	7 (23.3)	6 (25.0)	1 (16.7)	1.000
Preoperative antiplatelets, *n* (%)	10 (33.3)	8 (33.3)	2 (33.3)	1.000
Hyperlipidemia, *n* (%)	9 (30.0)	6 (25.0)	3 (50.0)	0.329
Hypertension, *n* (%)	12 (40.0)	10 (41.7)	2 (40.0)	1.000
PCA involvement, *n* (%)	6 (20.0)	5 (20.8)	1 (16.7)	1.000
Suzuki's stage (≤ 3), *n* (%)	23 (76.7)	19 (79.2)	4 (66.7)	0.603
mRS at discharge, mean ± SD	1.10 ± 0.31	1.08 ± 0.28	1.17 ± 0.408	0.559

Abbreviations: BMI, body mass index; CHS, cerebral hyperperfusion syndrome; IQR, interquartile range; MMD, moyamoya disease; PCA, posterior cortical atrophy.

^a^ Calculated by Fisher's exact test or unpaired *t*‐test, two‐tailed.

^b^ BMI was calculated as weight in kilograms divided by height in meters squared.

**TABLE 2:** Hemodynamic Baseline Measured by SAGSD.

Hemodynamic Parameters	All MMD Patients (*n* = 30)	Patients without CHS (*n* = 24)	Patients with CHS (*n* = 6)	*p* ^a^
PSV, mean ± SD	5.17 ± 0.45	5.16 ± 0.45	5.22 ± 0.5	0.781
EDV, mean ± SD	2.13 ± 0.18	2.15 ± 0.2	2.06 ± 0.06	0.067
VTI, mean ± SD	1.74 ± 0.13	1.74 ± 0.14	1.73 ± 0.08	0.931
TAMean, mean ± SD	3.01 ± 0.18	3.01 ± 0.16	3.08 ± 0.08	0.163
PI, mean ± SD	0.98 ± 0.16	0.97 ± 0.16	1.02 ± 0.17	0.566
RI, mean ± SD	0.57 ± 0.04	0.57 ± 0.04	0.59 ± 0.04	0.207
S/D, mean ± SD	2.44 ± 0.25	2.41 ± 0.24	2.55 ± 0.29	0.303

Abbreviations: CHS, cerebral hyperperfusion syndrome; EDV, end‐diastolic velocity; MMD, moyamoya disease; PI, pulsatility index; PSV, peak systolic velocity; RI, resistive index; S/D, systolic‐to‐diastolic ratio; SAGSD, synchronous arbitrary gate spectra Doppler; VTI, velocity‐time integral.

^a^ Calculated by an unpaired *t*‐test with Welch correction, two‐tailed.

### 3.2 SAGSD Morphological Characteristics of CHS and Quantitative Assessment

**FIGURE 3:**
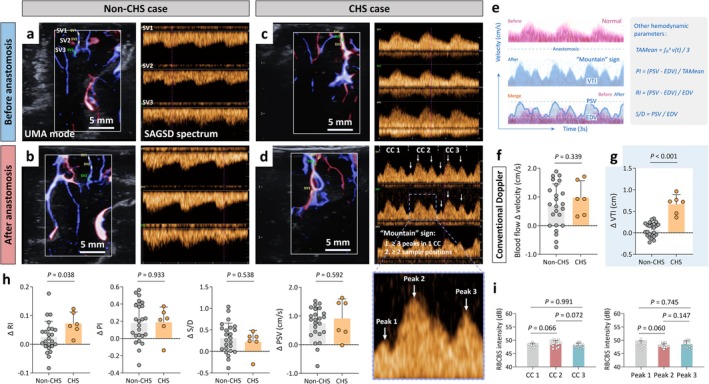
SAGSD phenotypic characteristics and quantitative description of the “mountain” sign. Representative images of SAGSD spectrum before (a, c) and after (b, d) anastomosis in revascularization of MMD patients. (e) Mechanistic schematic showing the relationship between spectral morphology and quantitative hemodynamic indices in a subcortical vessel (< 0.5 mm) that appeared “mountain” sign. Left: Pre‐ and post‐anastomotic velocity‐time waveforms (3‐s epochs); the typical “mountain” morphology appeared after anastomosis. The area under the curve denotes VTI. Superimposed tracings (bottom) highlight the post‐operative augmentation. Right: Derived hemodynamic indices are tabulated to quantify the spectral changes. (f) The average difference of blood flow velocity before and after anastomosis (Δ velocity) in patients with and without CHS measured by conventional Doppler US. The average of (g) velocity‐time integral change (Δ VTI), (h) peak systolic velocity change (Δ PSV), resistive index change (Δ RI), pulsatility index change (Δ PI), and systolic‐to‐diastolic ratio change (Δ S/D) before and after anastomosis in patients with and without CHS measured by SAGSD. *p*‐values were calculated by an unpaired *t*‐test with Welch correction, two‐tailed, for (i) RBCBS intensity among peaks within a single cardiac cycle and for each peak across successive cardiac cycles in five CHS patients (three SVs for each patient) who developed a post‐bypass “mountain” sign (each data point represents RBCBS intensity corresponding to a different sampling point [each peak]). *p*‐values were calculated by paired *t*‐test, two‐tailed. CC, cardiac cycle; CHS, cerebral hyperperfusion syndrome; MMD, moyamoya disease; RBCBS, red‐blood‐cell backscatter; SAGSD, synchronous arbitrary‐gate spectral Doppler; SV, sampling volume; UMA, ultra‐microangiography.

All intraoperative imaging measurements were successfully conducted before and after anastomosis, and no perioperative adverse events due to imaging examination were observed. The morphological characteristics of the SAGSD spectrum were evaluated postoperatively for all thirty patients. Postoperative SAGSD spectra remained largely unchanged relative to pre‐bypass recordings (Figure 3a,b). However, a specific morphological characteristic, termed the “mountain” sign, was identified in the SAGSD spectral waveforms after anastomosis in five out of six patients (83.3%) who later developed CHS and in none of the 24 patients without CHS (Fisher's exact test, two‐tailed, *p* < 0.05). This yielded a sensitivity of 83.3% (95% CI 36.1%–99.7%) and specificity of 100% (95% CI 85.8%–100%). The “mountain” sign is defined as the presence of three or more discrete velocity peaks (criterion 1) within a single cardiac cycle, with a shape resembling the mountain, and the simultaneous occurrence of this pattern at two or more sampling sites (criterion 2) on a continuous SAGSD flow‐time spectrum (Figure 3c,d). Following this, a mechanistic schematic of hemodynamic parameter differences is presented in Figure 3e, illustrating the relationship between spectral morphology and quantitative indices in subcortical vessels. Kappa value of the “mountain sign” between two observers was 0.760 (*p* = 0.006, 95% CI: 0.504–1.000) with observed agreement of 93.33%. The “mountain” sign proportion difference, Cohen's h = 2.30, yielded approximately 99.9% power (two independent proportions test, α = 0.05, two‐sided).

Sampling depth remained invariant across the MMD patients: Conventional Doppler US yielded 0.63 cm (IQR, 0.58–0.67) pre‐anastomosis and 0.64 cm (IQR, 0.60–0.67) post‐anastomosis, showing no significant difference (*p* = 0.820). Similarly, no significant change in SAGSD sampling depth was observed (*p* = 0.788). Between‐group comparison revealed no significant differences in velocity change (Δ velocity) by conventional Doppler (Figure 3f), while Δ VTI was significantly higher in CHS patients: 0.66 cm (IQR, 0.43–0.82) vs. 0.18 cm (IQR, 0.03–0.20) in non‐CHS patients (median Δ +0.48 cm [95% CI, 0.31 to 0.65]; *p* < 0.001, Figure 3g). Post hoc power analyses confirmed high achieved power for the key endpoints despite the limited sample size. For the between‐group difference in ΔVTI, the observed effect size (Cohen's d = 3.43) provided > 99.9% power (α = 0.05, two‐tailed). Other SAGSD‐derived hemodynamic parameters, such as Δ PSV, Δ RI, Δ PI, or Δ S/D, between patients who did and did not develop CHS (all *p* > 0.050, Figure 3h).

To assess the spectral homogeneity of the peaks defined in the “mountain” sign, Doppler energy was measured. This energy was quantified as the echo intensity derived from RBCBS for each peak of the “mountain” sign. In the spectral analysis of five CHS patients who presented with the “mountain” sign, two key observations emerged regarding RBCBS intensity: First, the intensity among peaks within a single cardiac cycle; second, the intensity of any specific peak across successive cardiac cycles. Both exhibited remarkable consistency. Specifically, no significant differences were detected in the RBCBS intensity either among peaks within a single cardiac cycle or for any given peak across successive cardiac cycles (Figure 3i).

### 3.3 Hemodynamic Evaluation of CHS Patients: Surface vs. Subcortical Parenchyma

**FIGURE 4:**
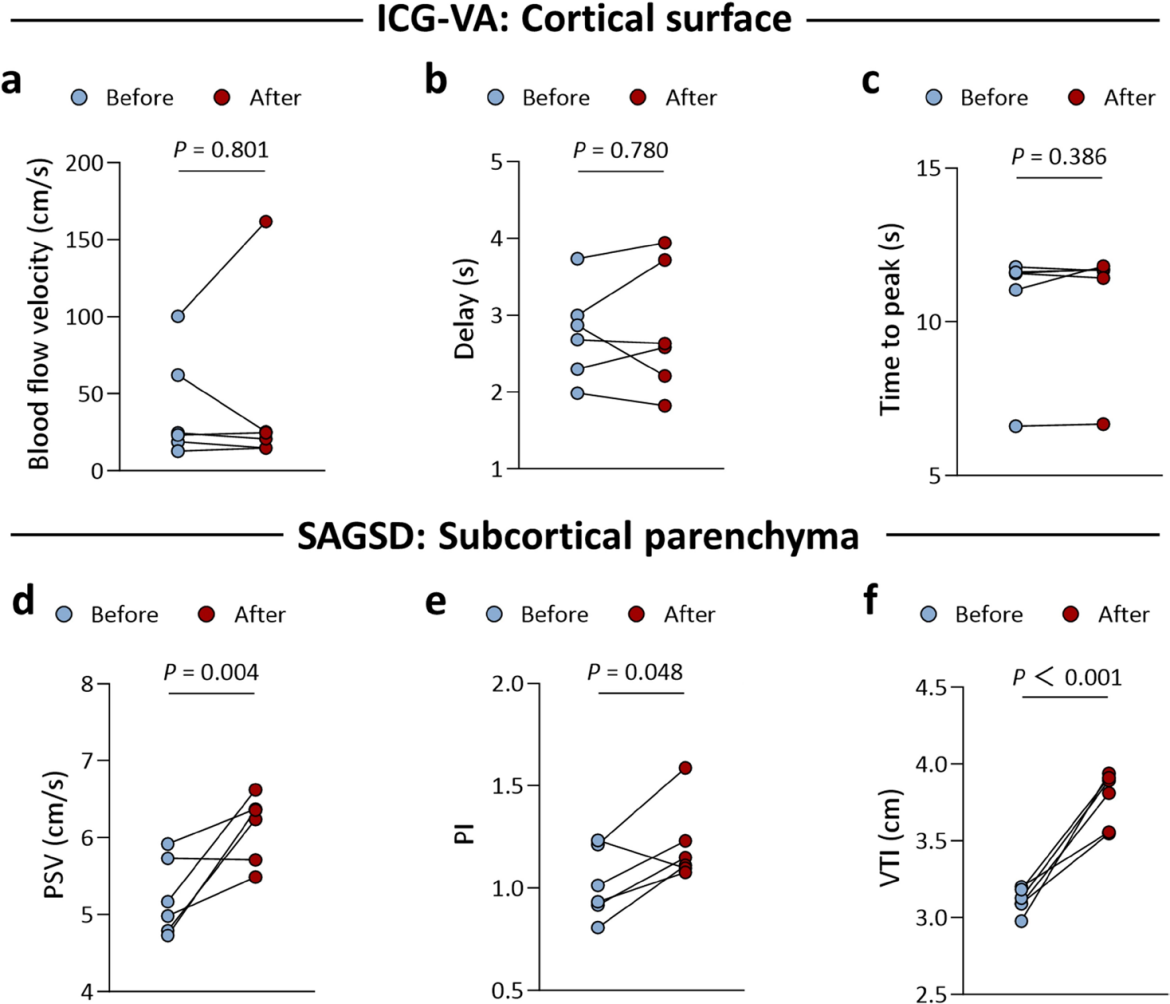
Evaluation of hemodynamic changes in post‐anastomosis CHS Patients using ICG‐VA vs. SAGSD. Cortical hemodynamics on the brain surface were quantitatively assessed with ICG‐VA in six CHS patients before and after anastomosis; no statistically significant alterations were observed in (a) blood flow velocity, (b) delay, or (c) time to peak. In contrast, quantitative evaluation of subcortical microvasculature with SAGSD revealed significant post‐bypass changes in (d) peak systolic velocity (PSV), (e) pulsatility index (PI), and (f) velocity‐time integral (VTI). *p*‐values were calculated using a two‐tailed, paired *t*‐test. CHS, cerebral hyperperfusion syndrome; ICG‐VA, indocyanine green videoangiography; SAGSD, synchronous arbitrary‐gate spectral Doppler.

**FIGURE 5:**
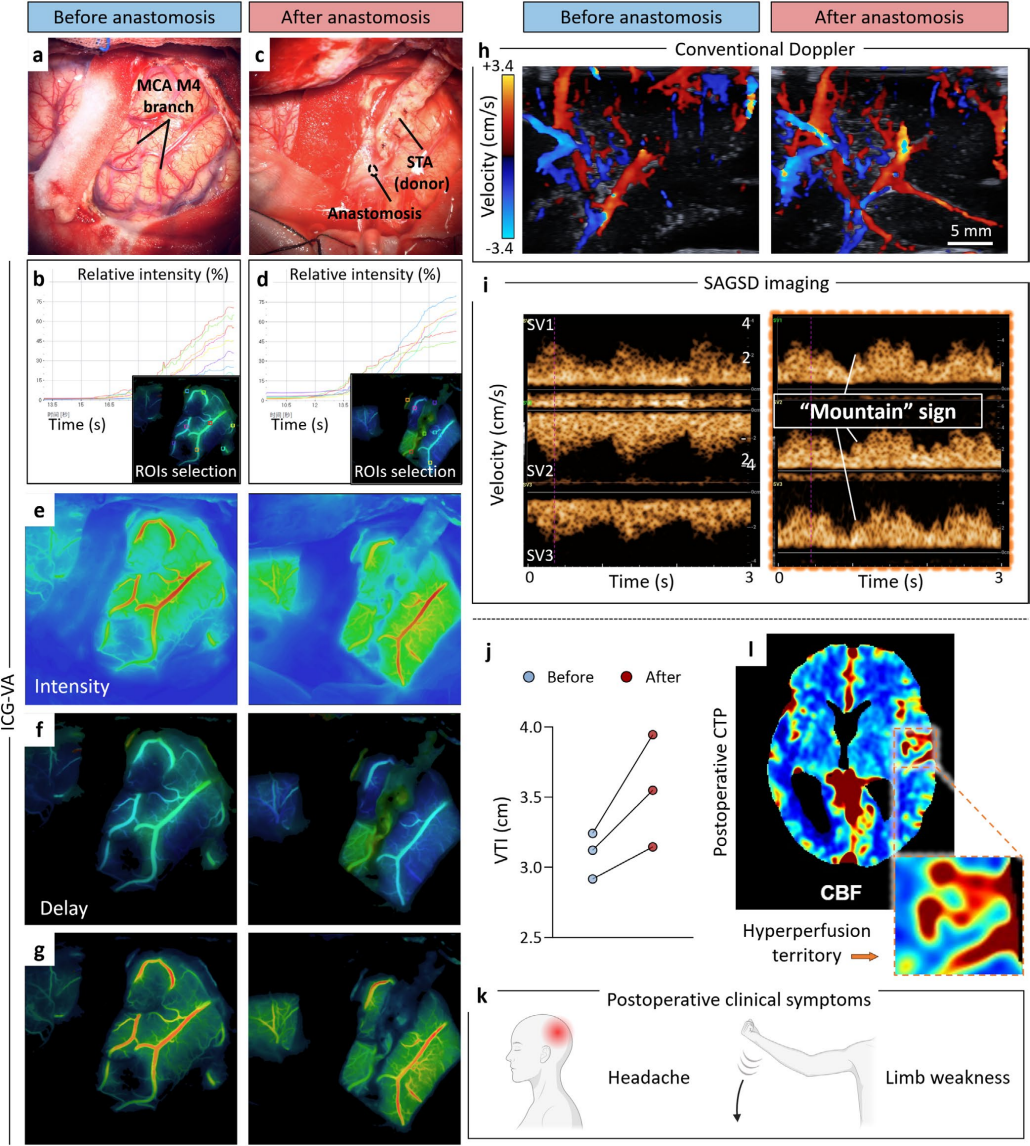
A representative case of evaluating intraoperative hemodynamics in CHS patients using different modalities. A 49‐year‐old woman with moyamoya disease underwent STA‐MCA bypass (a, c). The time–intensity curves of ICG‐VA (b, d) intensity (e), delay (f), and time to peak (g) maps of the brain surface indicated unchanged after anastomosis, and conventional Doppler merely demonstrated augmented flow signals (h). SAGSD interrogation of subcortical hemodynamics, however, revealed the characteristic “mountain” spectral pattern post‐revascularization (i), with velocity‐time integral (VTI) rising from 3.09 (mean) to 3.55 (mean) cm (j). CBF mode of postoperative CTP disclosed a focal hyperperfusion territory (highlighted and enlarged) beneath the left frontal cortex (k), indicating CHS. On post‐operative day 2, this patient developed a headache and contralateral limb weakness (l). Created with BioRender.com. CBF, cerebral blood flow; CHS, cerebral hyperperfusion syndrome; CTP, computed tomography perfusion; ICG‐VA, indocyanine green videoangiography; ROI, region of interest; SAGSD, synchronous arbitrary gate spectral Doppler; STA‐MCA, superficial temporal artery to middle cerebral artery; SV, sample volume.

Mean SAGSD sampling depths of CHS patients before and after anastomosis were 0.63 (IQR, 0.58–0.67) cm and 0.64 (IQR, 0.60–0.67) cm, respectively, showing no significant difference (*p* > 0.050). provides a detailed, quantitative comparison of perioperative hemodynamic parameters in patients with CHS as assessed by the two imaging modalities. Among the six CHS patients, intraoperative ICG‐VA demonstrated no significant post‐anastomotic alterations in average cortical blood flow velocity (Δ +2.5 cm/s [95% CI, −30.09 to 37.03]), delay (Δ + 0.1 s [95% CI, −0.43 to 0.55]), or time to peak (Δ +0.1 s [95% CI, −0.22 to 0.48]) (all *p* > 0.050, Figure 4a–c). Conversely, SAGSD spectrum‐derived parameters of subcortical vasculature revealed marked, consistent increments: PSV increased from 5.22 cm/s (IQR, 4.78–5.78) to 6.29 cm/s (IQR, 6.05–6.64) (median Δ +1.08 cm/s [95% CI, 0.54–1.61]; *p* = 0.004), RI from 0.59 (IQR, 0.56–0.64) to 0.67 (IQR, 0.64–0.70) (median Δ +0.18 [95% CI, 0.03–0.11]; *p* = 0.007), and VTI from 3.11 cm (IQR, 3.06–3.19) to 3.78 cm (IQR, 3.55–3.92) (median Δ +0.67 cm [95% CI, 0.43–0.90]; *p* < 0.001, Figure 4d–f). Figure 5 further illustrates an example of hemodynamic evaluation in a patient with CHS using different intraoperative techniques, including ICG‐VA, conventional Doppler, and SAGSD.

## 4 Discussion

In this preliminary single‐center study, we first assessed different intraoperative imaging modalities, including conventional Doppler US, SAGSD, and ICG‐VA, to evaluate the cerebral perfusion of MMD adults before and immediately after the anastomosis of STA‐MCA bypass (Figure 1). Based on the intraoperative data collection and hemodynamic assessment of 30 bypass surgeries, we believe that SAGSD imaging, compared with conventional Doppler US, has outstanding advantages such as high throughput, multi‐point sampling, no need for exogenous contrast agent injection, and high repeatability. By leveraging the UMA imaging modality, the system achieved a vascular detection sensitivity down to 0.1 mm. During the subsequent perioperative observation, six cases (20.0%) of patients developed CHS (Table 1), which was similar to the incidence of CHS in other studies [6, 7, 14, 18, 19, 22]. Moreover, the “mountain” sign was discovered on the SAGSD spectrum after vascular anastomosis in five of the CHS cases (Figure 3d).

Owing to the exuberant neovascularization in MMD, the multi‐peak appearance of the “mountain” sign within a single cardiac cycle was initially suspected to result from superimposed signals arising from distinct small vessels [4, 5, 29, 30]. However, because SAGSD simultaneously interrogates multiple loci within the same vascular segment, we observed similar, identical multi‐peak spectra at all sampled positions (the second criterion of the “mountain” sign). This spatial concordance indicates that the distinctive waveform morphology arises from a single vessel rather than from overlapping signals of separate small vessels. To further elucidate the homogeneity among these peaks, the raw data of Doppler energy were then extracted corresponding to each peak as a surrogate for the RBCBS intensity [31, 32]. Across the five patients, RBCBS intensity showed no significant difference either (i) at a single sampling site across successive cardiac cycles or (ii) among simultaneously sampled sites within the same cardiac cycle (Figure 3i). This congruence of spatial location and temporal sequence suggests that every peak contributing to the “mountain” sign of different SAGSD sampling volumes arises from the same blood vessel.

**SCHEME 1:**
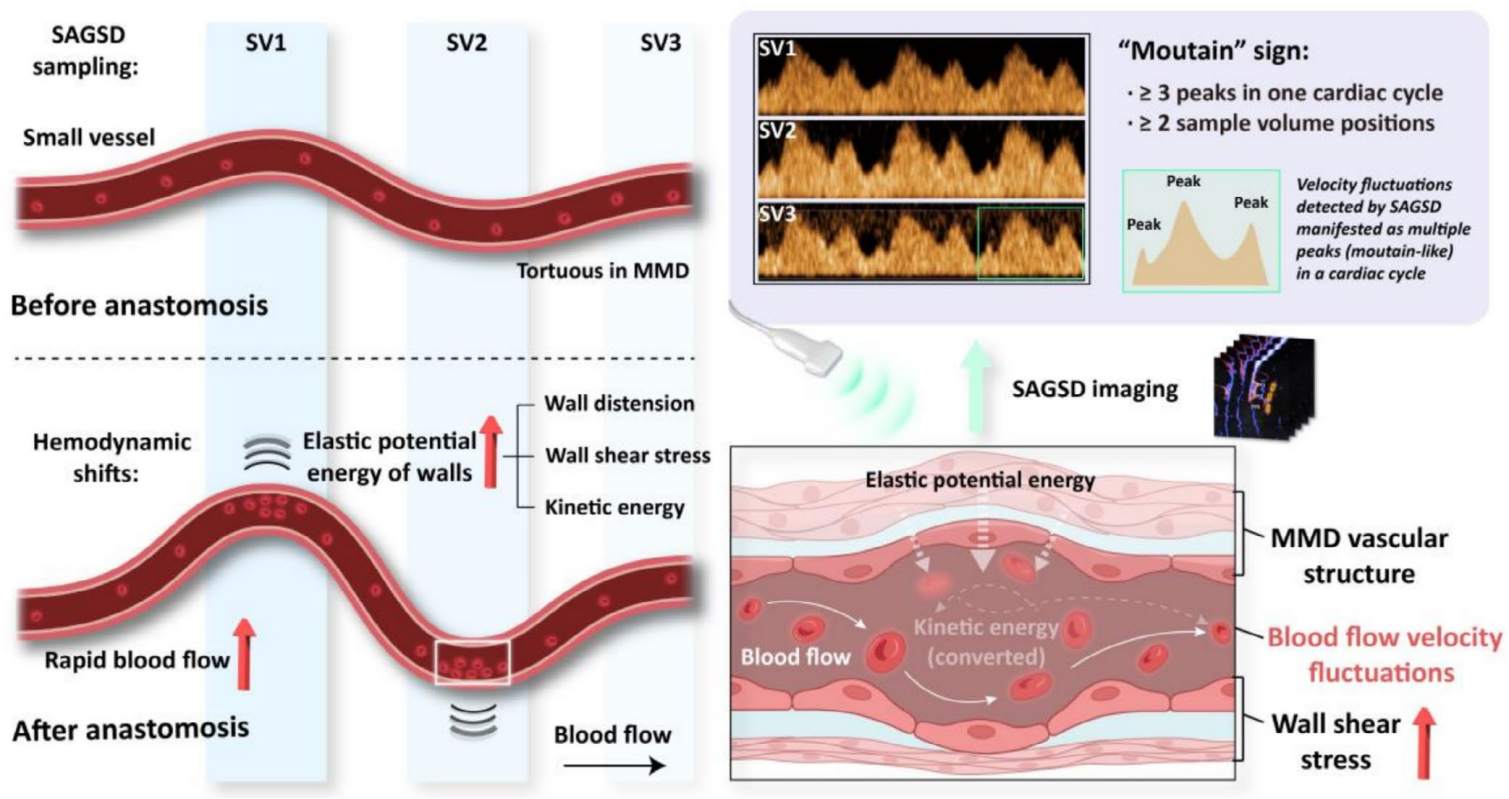
Illustration of the possible mechanism of the “mountain” sign after direct revascularization in MMD patients. After STA‐MCA anastomosis, abrupt flow augmentation within the fragile, densely arborized perforating network characteristic of moyamoya disease precipitates focal hemodynamic disruption. Within tortuous segments, the surge in blood flow increases elastic potential energy stored in the vessel wall; excess kinetic energy dissipates through (1) wall distension (2), elevated wall shear stress imposed by fibromuscular intimal hyperplasia, and (3) conversion to kinetic energy that causes velocity fluctuations. On SAGSD spectrograms, these fluctuations manifest as multiple irregular velocity peaks within a single cardiac cycle, the first spectroscopic criterion of the “mountain” sign. Created with BioRender.com. SAGSD, synchronous arbitrary gate spectral Doppler; MMD, moyamoya disease; SV, sample volume; CHS, cerebral hyperperfusion syndrome.

Based on these observations, we propose a mechanistic model for the genesis of the “mountain” sign (Scheme 1). After STA‐MCA anastomosis, abrupt flow augmentation within the fragile, densely arborized network of thin‐walled perforating vessels characteristic of MMD precipitates focal hemodynamic disruption [15, 33]. Within tortuous spiral segments, the sudden surge in local blood flow rapidly augments elastic potential energy stored in the vessel wall; excess kinetic energy is dissipated through (1) wall distension (2), elevated energy of wall shear stress (WSS) imposed by fibromuscular intimal hyperplasia, and (3) converted into kinetic energy that causes velocity fluctuations. On SAGSD spectrograms, these fluctuations manifest as multiple irregular velocity peaks within a single cardiac cycle, which is the first spectroscopic criterion of the “mountain” sign. The flow surge is quantitatively captured by a commensurate rise in VTI (Figure 3g), which represents the integral of the blood flow velocity curve over time. Sustained WSS elevation further increases endothelial permeability, predisposing to vasogenic edema and potential neurologic injury [7, 13, 34, 35]. Therefore, the presence of the “mountain” sign after revascularization may be an early hemodynamic indicator of CHS. The severity of hemodynamic shifts is directly observable: Yu et al. reported that venous arterialization marks profound flow alteration by ICG‐VA in one CHS patient and implicates it in CHS onset [22]. Although venous arterialization has been rarely observed in MMD patients undergoing direct revascularization in the past, this scarcity might be attributed to the depth limitations of ICG‐VA. Specifically, ICG‐VA is capable of evaluating only the surface of brain tissue and lacks information on the hemodynamics of deep brain tissue [36, 37]. In addition, current imaging evidence, such as CTP, is predominantly assessed based on increased subcortical brain tissue perfusion [38, 39, 40]. Coupled with the low‐velocity, low‐resistance spectral signature we document, these observations indicate that the “mountain” sign most likely reflects post‐revascularization instability mediated by arterialized subcortical veins, which might be the tributaries of basal dural venous sinuses according to anatomical position.

Although our findings are encouraging, several limitations merit consideration. First, the retrospective design inherently carries a risk of selection bias. Second, although the observed effect sizes for both ΔVTI changes and the “mountain” sign were very large (Cohen's d = 3.43 and Cohen's h = 2.30, respectively), yielding achieved post hoc power > 99.9% for detecting these differences, we fully recognize that the modest sample size remains a key limitation of this exploratory study. Small cohorts can lead to overestimation of effect magnitudes and reduced precision in confidence intervals, particularly for the sensitivity estimate of the “mountain” sign. Larger, prospective, multicenter cohorts are essential to confirm the diagnostic accuracy, generalizability, and clinical utility of SAGSD‐derived markers in predicting and managing CHS following revascularization in MMD. Third, in this study, to measure the hemodynamics of small blood vessels, the UMA mode of SAGSD interrogates small vessels whose exact pre‐ and post‐anastomotic identification is inherently uncertain. We therefore acquired triplicate measurements from three distinct subcortical arterioles at each time point and used the vessel‐wise mean for analysis. In addition, to alleviate depth‐related bias, we verified that the average sampling depth did not differ (with no significant statistical difference) before and after anastomosis. Finally, variability in SAGSD spectrum interpretation among the radiologists may have been a limitation in our study. Hence, further studies that evaluate inter‐reader agreement for SAGSD and possibly inter‐modality with ICG‐VA are needed.

## 5 Conclusion

In summary, synchronous arbitrary‐gate spectral Doppler advances intra‐operative hemodynamic assessment by delivering high‐resolution, multi‐site microvascular data without exogenous contrast. Among patients who later developed cerebral hyperperfusion syndrome (CHS), the majority exhibited the novel “mountain” spectral phenotype, quantitatively corroborated by a marked rise in velocity‐time integral. This sign may serve as an early predictor for CHS, providing a basis for timely intervention and improved surgical outcomes.

## Author Contributions

Conceptualization, H.D., W.N., and Z.L. Supervision, Y.G., C.G. Project administration, Z.F., R.W., Y.L., and X.Z. Funding acquisition, H.D. Formal analysis and methodology, X.H., X.Z., and Z.L. Resources and software, H.D., Y.G., and Z.L. Visualization, H.Y., and X.Z. Validation, H.J., J.S., and W.N. Writing – original draft, X.Z. and X.H. Writing – Review and Editing, H.D. and J.Y.

## Funding

This work was supported by the National Natural Science Foundation of China, 82272017.

## Ethics Statement

This study was approved by the Ethics Committee of Huashan Hospital, Fudan University (Approval No. 2021–724) and registered in ClinicalTrials.gov (ChiCTR2100050390), in accordance with the ethical principles of Good Clinical Practice and the Declaration of Helsinki. Written informed consent was obtained from all participants.

## Conflicts of Interest

The authors declare no conflicts of interest.

## Supporting information


**Table S1:** Intraoperative SAGSD and Conventional CDFI Modalities Settings. **Table S2:** Definitions of SAGSD‐derived Hemodynamic Parameters. **Figure S1:** A schematic workflow of the intraoperative imaging examination process of STA‐MCA bypass in MMD adults.

## Data Availability

The data that support the findings of this study are available from the corresponding author upon reasonable request.
