# Supplementary material for: Unusual Synchronous Arbitrary‐Gate Doppler Spectra Enable Intraoperative Hemodynamic Warning of Cerebral Hyperperfusion Syndrome on Moyamoya Disease
Source: CNS Neurosci Ther. 2026 Mar 11;32(3):e70829. doi: 10.1002/cns.70829 (PMC13093794; doi:10.1002/cns.70829)
Supplement: Supplementary file 1 — Table S1: Intraoperative SAGSD and Conventional CDFI Modalities Settings. Table S2: Definitions of SAGSD‐derived Hemodynamic Parameters. Figure S1: A schematic workflow of the intraoperative imaging examination process of STA‐MCA bypass in MMD adults. [file CNS-32-e70829-s001.docx]

# Supporting information

**Unusual Synchronous Arbitrary-Gate Doppler Spectra Enable Intraoperative Hemodynamic Warning of Cerebral Hyperperfusion Syndrome on Moyamoya Disease**

Xiandi Zhang^1#^, PhD; Wei Ni^2#^, MD; Xing Hu^1#^, MD; Heng Yang^2^, MD; Jiabin Su^2^, MD; Hanqiang Jiang^2^, MD; Chao Gao^2^, MD; Ruiyuan Weng^2^, PhD; Zhen Fan^2^, MD; Yiming Li^1^, MD; Jinhua Yu^3^, PhD; Zhaoling Lu^4*^, PhD; Yuxiang Gu^2*^, MD; Hong Ding^1,5*^, MD

^1^Department of Ultrasound, Huashan Hospital, Fudan University, Shanghai 200040, China.

^2^Department of Neurosurgery, Huashan Hospital, Fudan University, Shanghai 200040, China.

^3^ School of Information Science and Technology, Fudan University, Shanghai 200082, China.

^4^ Mindray MIS Innovation Center, 690 North McCarthy Blvd, Suite 220, Milpitas, CA 95035, USA.

^5^National Clinical Research Center for Aging and Medicine, Fudan University, Shanghai 200040, China.

^*^Corresponding authors:

[z.lu@mindray.com](mailto:z.lu@mindray.com) (Z.L.), 690 North McCarthy Blvd, Suite 220, Milpitas, CA 95035, USA

[guyuxiang1972@126.com](mailto:guyuxiang1972@126.com) (Y.G.), No.12 Middle Urumqi Road, Shanghai 200040, P. R. China

ding_hong@fudan.edu.cn (H.D.), No.12 Middle Urumqi Road, Shanghai 200040, P. R. China

^#^X.Z., W.N. and X.H. are contributed equally to this work.

# Supplementary Materials and Methods

### 1.1 Detailed steps of intraoperative imaging examination

During intraoperative conventional Doppler examination, after the bypass strategy has been determined, the L10-3 probe (Nuewa A20W, Mindray Bio-Medical Electronics Co., Ltd., Shenzhen, China) should be wrapped in a sterile sheath and positioned on the same area before and after the anastomosis near the bypass vessel. Consistency in the setting parameters is essential for each inspection. In each sonographic section, synchronous arbitrary-gate spectral Doppler (SAGSD) examination was performed subsequent to the conventional Doppler ultrasound examination.

For intraoperative indocyanine green videoangiography (ICG-VA) examination, a 10-mg bolus of ICG (ICG Pulsion, Pulsion Medical Systems) was peripherally injected intravenously followed by 10 ml of saline flush with the microscope kept still for at least 1 minute before and after anastomosis. While ensuring the full display of the bridging vessels, the imaging should, as far as possible, be maintained within the same field as that before the operation. FLOW 800 (Carl Zeiss AG, Oberkochen, Germany) software was utilized to assess the hemodynamics across four regions of interest (ROIs) chosen by the same surgeon. The consistency of each ROI across each patient was verified manually by the research team.

# Supplementary Tables

**Supplementary** **Table 1.** Intraoperative SAGSD and Conventional CDFI Modalities Settings

| **Parameters (abbreviation)** | **Unit** | **SAGSD** | **Conventional Doppler** |
| --- | --- | --- | --- |
| Frequency (F) | MHz | 5.0 | 5.0 |
| Gain (G) | dB | 86.0 | 80 |
| Wall filter (WF) | Hz | 96.0 | 79.0 |
| Pulse repetition frequency (PRF) | Hz | 1.2k | 1.0k |
| Depth (D) under parenchyma | cm | 0.5-2.5 | 0.5-2.5 |
| Mechanical index (MI) | - | 0.4 | 1.0 |
| Thermal index for soft (TIS) | - | 1.1 | 0.2 |
| Time gain compensation (TGC) | dB | all are in the middle | all are in the middle |

SAGSD, synchronous arbitrary gate spectra Doppler; US, ultrasound; CDFI, color doppler flow imaging; PD; power Doppler; CEUS, contrast-enhanced ultrasound.

**Supplementary** **Table 2.** Definitions of SAGSD-derived Hemodynamic Parameters

| **Hemodynamic Parameters** | **Definitions** | **Calculation method** |
| --- | --- | --- |
| PSV | Peak systolic velocity. The maximum blood flow velocity during the systolic phase of the cardiac cycle. | - |
| EDV | End-diastolic velocity. The blood flow velocity at the end of the diastole. | - |
| VTI | Velocity-time integral. The integral of the blood flow velocity curve over time. | *∫v(t)dt^c^* |
| TAMean | Time-averaged mean velocity. The average velocity over a complete cardiac cycle. | *∫v(t)dt / T^#^* |
| PI | Pulsatility index. A dimensionless parameter quantifying the variability of blood flow velocity throughout the cardiac cycle. | *(PSV - EDV) / TAMean* |
| RI | Resistive index. A dimensionless parameter used to assess downstream vascular resistance. | *(PSV - EDV) / PSV* |
| S/D | Systolic-to-diastolic ratio. The ratio of peak systolic to end-diastolic velocity. | *PSV / EDV* |

***^*^****v(t)* represents the function of blood flow velocity varying with time.

*^#^*T represents the time interval of measurement.

# Supplementary Figures

**
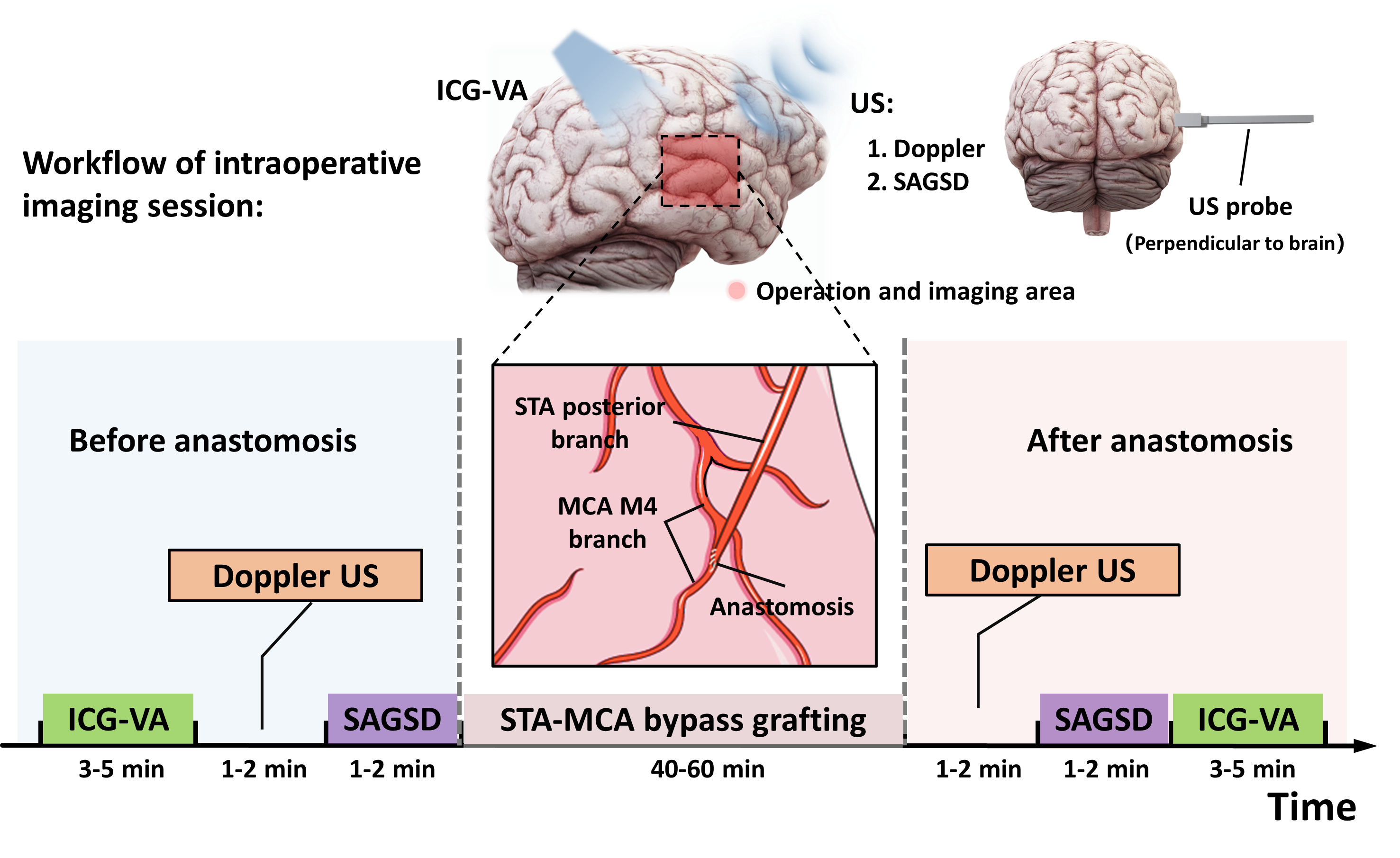
**

**Supplementary** **Figure 1.** A schematic workflow of intraoperative imaging examination process of STA-MCA bypass in MMD adults.

Abbreviations: ICG-VA, indocyanine green videoangiography; US, ultrasound; SAGSD, synchronous arbitrary gate spectra Doppler; STA, superficial temporal artery; MCA, middle cerebral artery.
